# Polyaminated, acetylated and stop codon readthrough of recombinant *Francisella tularensis *universal stress protein in *Escherichia coli*

**DOI:** 10.1371/journal.pone.0299701

**Published:** 2024-04-29

**Authors:** Benjamin Girardo, Lawrence M. Schopfer, Yinshi Yue, Oksana Lockridge, Marilynn A. Larson

**Affiliations:** 1 Pathology and Microbiology Department, University of Nebraska Medical Center, Omaha, NE, United States of America; 2 Eppley Institute, University of Nebraska Medical Center, Omaha, NE, United States of America; Pondicherry University, INDIA

## Abstract

Recombinant *Francisella tularensis* universal stress protein with a C-terminal histidine-tag (rUsp/His_6_) was expressed in *Escherichia coli*. Endogenous *F*. *tularensis* Usp has a predicted molecular mass of 30 kDa, but rUsp/His_6_ had an apparent molecular weight of 33 kDa based on Western blot analyses. To determine the source of the higher molecular weight for rUsp/His_6_, post translational modifications were examined. Tryptic peptides of purified rUsp/His_6_ were subjected to liquid chromatography tandem mass spectrometry (LC-MS/MS) and fragmentation spectra were searched for acetylated lysines and polyaminated glutamines. Of the 24 lysines in rUsp/His_6_, 10 were acetylated (K63, K68, K72, K129, K175, K201, K208, K212, K233, and K238) and three of the four glutamines had putrescine, spermidine and spermine adducts (Q55, Q60 and Q267). The level of post-translational modification was substoichiometric, eliminating the possibility that these modifications were the sole contributor to the 3 kDa extra mass of rUsp/His_6_. LC-MS/MS revealed that stop codon readthrough had occurred resulting in the unexpected addition of 20 extra amino acids at the C-terminus of rUsp/His_6_, after the histidine tag. Further, the finding of polyaminated glutamines in rUsp/His_6_ indicated that *E*. *coli* is capable of transglutaminase activity.

## Introduction

Bacteria, plants, archaea, fungi, and select invertebrates have universal stress proteins (Usp) whose proposed function is to protect against a variety of stressors (i.e., reactive oxygen species and starvation), assist in iron scavenging, and sense ion and energy levels [1, 2]. *Escherichia coli* and *Mycobacterium tuberculosis* contain 6 and 10 Usp isoforms, respectively, and associated studies have provided knowledge on the potential function of Usp [3–6]. However, the presence of multiple Usp isoforms has complicated data interpretation in these investigations. In contrast to other bacteria, *Francisella tularensis* contains a single copy, highly conserved *usp* gene. *F*. *tularensis* causes the zoonotic disease tularemia, which can be fatal if left untreated. A first step in our goal to elucidate the function of Usp in this intracellular pathogen was to express the recombinant Usp and produce antibodies for detection of Usp in *Francisella tularensis*.

*F*. *tularensis* Usp has a theoretical molecular weight of 30,188 Da calculated from the amino acid sequence (accession number WP_003021757). Recombinant *F*. *tularensis* Usp (rUsp/His_6_) has a C-terminal histidine tag comprised of LEHHHHHH, which contributes 1065 Da for a combined molecular weight of 31,253. However, Western blots probed with an antibody to *F*. *tularensis* Usp indicated that rUsp/His_6_ has an apparent molecular weight of 33 kDa, about 3 kDa heavier than predicted for this protein.

Since recombinant proteins frequently carry non-native post-translational modifications [7–9], a likely explanation for the excess mass of *F*. *tularensis* rUsp/His_6_ was post-translational modification by *E*. *coli*. For this reason, recombinant proteins intended for therapeutic use in humans are rigorously characterized for undesired modifications. Previous studies showed that 0.8% of the insulin Lispro protein expressed in *E*. *coli* was acetylated on lysine 31 of the B chain [8] and the recombinant human IL-10 protein was partly acetylated on 4 out of 13 lysines [9]. The *E*. *coli* expression system modified recombinant human histones and recombinant human heat shock protein HSP70 by adding putrescine, spermidine, and spermine [7]. When acetylated lysines lose their positive charge, a less soluble protein is often produced that is prone to aggregation [10]. Post-translational modifications are typically substoichiometric, so only a small percentage of the associated residues in a given recombinant protein are modified.

In view of these precedents, we searched for acetylated lysines and polyaminated glutamines in purified *F*. *tularensis* rUsp/His_6_ expressed in *E*. *coli*. Our study provides evidence that *F*. *tularensis* rUsp/His_6_ was acetylated on 10 out of 24 lysines and polyaminated on 3 out of 4 glutamines, but these modified peptides were found in low abundance, suggesting a frequency of less than 1% for a given residue. Therefore, the low frequency of post-translational modifications led us to search for an alternative explanation for the extra 3 kDa molecular weight of *F*. *tularensis* rUsp/His_6_. Mass spectrometry data identified the unexpected addition of 20 amino acids after the C-terminal histidine tag. Although stop codon readthrough has been shown to occur [11–14], this phenomenon in the pET expression system for recombinant protein production in *E*. *coli* has not been previously reported. The pET expression system was described more than thirty years ago [15] and remains one of the most popular systems for recombinant protein production. Improvements to the pET expression system have been made [16], but additional precautions are warranted to prevent stop codon readthrough.

## Materials and methods

Nickel-nitrilotriacetic acid (Ni-NTA) chromatography resin, Thermo Scientific #88221. Protein G agarose, Protein Mods LLC, IgG binding capacity 20 mg/mL #PGG. CNBr-activated Sepharose 4-Fast Flow. Amersham Biosciences #17-0981-01. C18 spin columns Pierce #89870. Steriflip vacuum filtration system 0.22 μm membrane Millipore #SCGP00525. Trypsin sequencing grade modified, Promega #V5113. Precast polyacrylamide gels Bio-Rad #4561044 (12%). Polyvinylidene fluoride (PVDF) 0.45 μm Immobilon-P transfer membrane, Millipore #IPVH00010. Enhanced Chemiluminescence (ECL) reagents Radiance Plus, Azure Biosystems #AC2103. Affinity purified rabbit anti-denatured Usp polyclonal, produced in-house. Donkey anti-rabbit IgG conjugated to horse radish peroxidase (HRP), Jackson ImmunoResearch #711-035-152. Bicinchoninic acid (BCA) protein assay, Pierce #23255. pET28a, Novagen #69864–3. 2x Yeast extract tryptone culture medium (2YT), Sigma #Y2377.

### Bacterial strains and culturing

*F*. *tularensis* LVS (NR-28537) was obtained from BEI Resources. *E*. *coli* BL21(DE3) was purchased from Novagen. *F*. *tularensis* was subcultured on chocolate agar plates (Remel) for 2 days at 37°C and then used to inoculate filter-sterilized Chamberlain’s Chemically Defined Medium (CDM). Chamberlain’s medium contains 40 mg spermine phosphate per liter [17]. *F*. *tularensis* LVS was grown at 37°C in CDM to mid-log growth phase. *E*. *coli* BL21(DE3) containing pET28a expressing *F*. *tularensis* rUsp/His6 was grown in 2YT at 37°C to an optical density at 600 nm of approximately 1, prior to induction with 0.5 mM IPTG at 18°C for 20 h. To check the solubility and approximate yield of rUsp/His_6_, an aliquot containing 25 x 10^10^ of bacterial cells was lysed by sonication in 25 mL of 25 mM Tris HCl, 100 mM NaCl, 20 mM sodium fluoride, 55 mM sodium ortho-vanadate, 55 mM pyrophosphate, 1 mM PMSF, and 2 μg/mL aprotinin. The cell suspension was clarified by centrifugation at 4000 rpm (2683xg) for 30 min in a table top Sorvall centrifuge cooled to 10˚C. Fine particles were removed by filtration through a 0.22 μm membrane. The final protein concentration was 3 mg/mL based on a BCA assay.

### Cloning of recombinant *F*. *tularensis* Usp into *E*. *coli* expression plasmid

The *usp* gene (locus tag FTT-RS01275) encoding the universal stress protein from *F*. *tularensis* SCHU S4 was amplified using PCR with the forward primer 5’-TCTCCATGGCGTACAAAAAGGTTTTATTAGC and reverse primer 5’-ATCTCTCGAGTTTAAGCCTTACAACCAAAACATC. The resulting amplicon was digested with NcoI and XhoI restriction endonucleases and cloned into the respective sites in the pET28a expression plasmid. This cloning design was expected to produce a recombinant *F*. *tularensis* Usp with a C-terminus histidine tag, which adds 8 residues (LEHHHHHH) to the 278 amino acid protein for affinity purification. The DNA sequence of the *F*. *tularensis usp* gene in pET28a was confirmed before the plasmid was transfected into *E*. *coli* BL21(DE3) for expression. To ensure that *F*. *tularensis usp* which was cloned into pET28a would produce the correct amino acid sequence, Sanger sequencing of this expression plasmid was performed in both directions with appropriate primers, by the UNMC Genomics Core Facility.

### Purification of recombinant *F*. *tularensis* Usp with C-terminal histidine tag

After induction of the pET vector for the expression of *F*. *tularensis* rUsp/His_6_ in *E*. *coli* BL21(DE3), bacterial cells were washed in PBS and lysed in 120 mL of 20 mM sodium phosphate, 0.5 M NaCl, 30 mM imidazole (pH 7.4) by passage through an EmulsiFlex-C3 (Avestin). The lysate was clarified by centrifugation in a Sorvall centrifuge for 30 min at 2683xg, followed by filtration through a 0.22 μm membrane. *F*. *tularensis* rUsp/His_6_ was purified by Ni-NTA chromatography by gravity flow through 15 mL Ni-NTA packed into Pharmacia column C16/20. The Ni-NTA column was equilibrated with 100 mL binding buffer (20 mM sodium phosphate, 0.5 M NaCl, 30 mM imidazole, pH 7.4) before the clarified lysate was manually loaded. Material that did not bind was washed off with 150 mL binding buffer. Washing was complete when the absorbance at 280 nm of the effluent was the same 0.085 as the absorbance of the binding buffer. Bound protein was eluted with elution buffer (20 mM sodium phosphate, 0.5 M imidazole, 0.5 M NaCl pH 7.4). Four mL fractions were collected manually and tested for Usp by Western blot. The highest concentration of Usp was in fractions 3–5. Pooled fractions were dialyzed against 4 L of phosphate buffered saline (pH 7.4) at 4˚C to remove imidazole and to reduce the salt concentration. Twenty mg of purified *F*. *tularensis* rUsp/His_6_ was recovered from 200 mL culture.

### Production of polyclonal antibodies to denatured *F*. *tularensis* rUsp/His_6_ in rabbits

Purified *F*. *tularensis* rUsp/His_6_ in phosphate buffered saline (pH 7.4) was turbid at 2 mg/mL, but almost clear at 0.5 mg/mL. A 10 mL solution of 0.5 mg/mL *F*. *tularensis* rUsp/His_6_ in phosphate buffered saline was heated in a boiling water bath for 10 min to denature the protein. The denatured *F*. *tularensis* rUsp/His_6_ solution was sent to Pacific Immunology (Ramona, CA) for injection into two New Zealand White rabbits. Each rabbit was immunized five times with antigen in Incomplete Freund’s Adjuvant over a period of three months. Serum was collected on day 49 and at 14-day intervals thereafter. A total of 313 mL of serum was collected.

### Affinity purification of antibodies to denatured *F*. *tularensis* rUsp/His_6_ from rabbit serum

The antiserum (313 mL) was clarified by centrifugation, and IgG was enriched by ammonium sulfate precipitation [18]. The ammonium sulfate pellet was suspended in 140 mL of 10 mM Tris HCl (pH 8), 0.02% azide and desalted by dialysis against 4 x 4 liters of 10 mM Tris HCl (pH 8), 0.1% azide at 4°C. The dialyzed protein had a volume of 172 mL. Total IgG was isolated on a 30 mL column of Protein G agarose. After the desalted protein suspension was applied to the Protein G column, the column was washed with 200 mL of 10 mM Tris HCl (pH 8). Bound protein was eluted with 0.1 M glycine (pH 2.7). Three mL fractions were collected into tubes preloaded with 0.3 mL of 1 M Tris HCl (pH 8). The eluted samples were immediately mixed to raise the pH of the eluted proteins from pH 2.7 to 8. The total amount of rabbit IgG collected was 430 mg.

Antibodies to denatured *F*. *tularensis* rUsp/His_6_ were separated from total rabbit IgG by affinity chromatography to immobilized rUsp/His_6_. Immobilization was performed by coupling 4 mg of denatured, purified rUsp/His_6_ to 3 mL of swollen CNBr-activated Sepharose beads. Eighteen mg of affinity purified rabbit antibodies to denatured *F*. *tularensis* rUsp/His_6_ was recovered from 430 mg of total rabbit IgG (4%).

### Western blot

*F*. *tularensis* lysate and purified rUsp/His_6_ were separated on 12% precast SDS gels (Bio-Rad). Proteins were transferred from the polyacrylamide gel to 0.45 μm PVDF membrane on a Bio-Rad Trans-Blot SP semi-dry transfer cell. Blots were hybridized with 5 μl (3.5 μg) affinity purified rabbit polyclonal antibodies to denatured *F*. *tularensis* rUsp/His_6_ in 20 mL Tris buffered saline containing 5% dry milk, overnight at 4°C. The secondary antibody, donkey anti-rabbit IgG conjugated to horse radish peroxidase, was diluted 2 μL (0.4 mg/mL in 50% glycerol) into 20 mL Tris buffered saline, 5% dry milk. Washed blots were developed with ECL reagents from Azure Bioimaging and images were acquired on the Azure 600 Bioimaging device.

### Experimental design and statistical rationale

A total of 29 Orbitrap data sets for *F*. *tularensis* rUsp/His_6_ were searched for acetylated and polyaminated adducts using Protein Prospector software. Data sets typically covered 92 to 98.9% of the amino acid sequence of the Usp protein. We manually evaluated each candidate MS/MS spectrum and found convincing evidence for acetylated and polyaminated peptides in 6 Orbitrap data sets. We rejected potential adducts in 23 data sets because the MS/MS spectra did not convince us that an adduct was the only possible interpretation of the data.

We required the MS/MS spectrum to contain ions that identify the modified residue. We searched for alternative interpretations of the MS/MS data and rejected candidate adducts that could be explained by substituting amino acids for the presumed adduct. For example, 128 Da spermidine could also represent 128 Da lysine; 71 Da putrescine could also represent 71 Da Alanine. The substituted amino acids had to fit the known amino acid sequence of *F*. *tularensis* Usp. Examples of rejected candidate adducts are described in the Discussion section and include an MS/MS spectrum of a false positive.

### Sample preparation for mass spectrometry

Samples were digested with trypsin either in solution or in gel slices. Our protocol for in solution digestion is to reduce disulfide bonds with 10 mM dithiothreitol, alkylate with 50 mM iodoacetamide, digest with trypsin overnight at 37°C, and desalt on a C18 spin column.

The in-gel protocol for the analysis of *F*. *tularensis* Usp begins by cutting out a Coomassie blue stained gel slice migrating in the 30 to 35 kDa range. The gel slice is destained with 50% acetonitrile, dried, reduced with dithiothreitol in 100 mM ammonium bicarbonate (pH 8), alkylated with iodoacetamide, washed with 50% acetonitrile, and dried in a vacuum centrifuge [19]. Protein in the gel slice is digested with trypsin in 100 mM ammonium bicarbonate (pH 8) overnight at 37°C. Peptides are extracted with 50% acetonitrile, 0.1% TFA, and dried. The peptides are cleaned on a C18 spin column, dried, and dissolved in 20 μl water.

Protein concentration in the peptide solution was estimated from absorbance at 280 nm in a Nanodrop spectrophotometer (Thermo Fisher Scientific). An absorbance at 280 nm of 1 corresponds to a protein concentration of 1 μg/μl. Injection of 1 μl peptide solution at 1 μg/μl protein concentration yielded 99% coverage of *F*. *tularensis* Usp when evaluating purified rUsp/His_6_.

### Liquid chromatography-tandem mass spectrometry

Peptide separation was performed with a Thermo RSLC Ultimate 3000 ultra-high pressure liquid chromatography system (Thermo Scientific) at 36°C, as previously described [20]. Solvent A was 0.1% formic acid in water, and solvent B was 0.1% formic acid in 80% acetonitrile. Peptides were loaded onto an Acclaim PepMap 100 C18 trap column (75 μm x 2 cm; Thermo Scientific catalog number 165535) at a flow rate of 4 μL/min and washed with 98% solvent A/2% solvent B for 10 minutes. Desalted peptides were pumped into a Thermo Easy-Spray PepMap RSLC C18 column (75 μm x 50 cm with 2 μm particles, Thermo Scientific catalog number ES803) and separated at a flow rate of 300 nL/min using a gradient of 9 to 50% solvent B in 30 min, 50 to 99% solvent B in 40 min, hold at 99% solvent B for 10 min, 99 to 9% solvent B in 4 min, and hold at 9% solvent B for 16 min.

Eluted peptides were sprayed directly into a Thermo Orbitrap Fusion Lumos Tribrid mass spectrometer (Thermo Scientific). Data were collected using data dependent acquisition. A survey full scan MS (from 350–1800 m/z) was acquired in the Orbitrap with a resolution of 120,000. The AGC target (Automatic Gain Control for setting the ion population in the Orbitrap before collecting the MS) was set at 4 x 10^5^ and the ion filling time was set at 50 msec. The 25 most intense ions with charge state of 2–6 were isolated in a 3 sec cycle and fragmented using high-energy collision induced dissociation with 35% normalized collision energy. Fragment ions were detected in the Orbitrap with a mass resolution of 30,000 at 200 m/z. The AGC target for MS/MS was set at 5 x 10^4^, and dynamic exclusion was set at 30 sec with a 10 ppm mass window. Data were reported in *.raw format. The *.raw data files were converted to *.mgf files using MSConvert, a ProteoWizard Tool from SourceForge.

### Protein prospector search for modified peptides

The search parameters on the Batch-Tag Web page in Protein Prospector (prospector.ucsf.edu/prospector/mshome.htm) were as follows. Database: User protein. User Protein Sequence: pasted the FASTA file of protein WP_003021757 into the window for User Protein Sequence. Taxonomy: *Francisella tularensis*. Precursor charge range: 2, 3, 4, 5. Parent Tol: 20 ppm, Frag Tol: 30 ppm. Instrument: ESI-Q-high-res. Digest: No enzyme. (Note that the no enzyme digestion option is justified by the overnight tryptic digestion, which provides ample opportunity for non-specific cleavage.) Max Missed Cleavages: 2. Constant mods: none. Expectation Calc Method: None. Variable Mods: Carbamidomethyl (C), Oxidation (M). User Defined Variable Modifications: Acetyl K. C2O1H2; mass modification range 41.9 to 42.1. Putrescine Q. C4H9N1; mass modification range 70.9 to 71.3. Spermidine Q. C7N2H16; mass modification range 127.9 to 128.4. Spermine Q. C10H23N3; mass modification range 185.0 to 185.4.

## Results

### Immunoblot comparison of endogenous *F*. *tularensis* Usp and rUsp/His_6_

**Fig 1:**
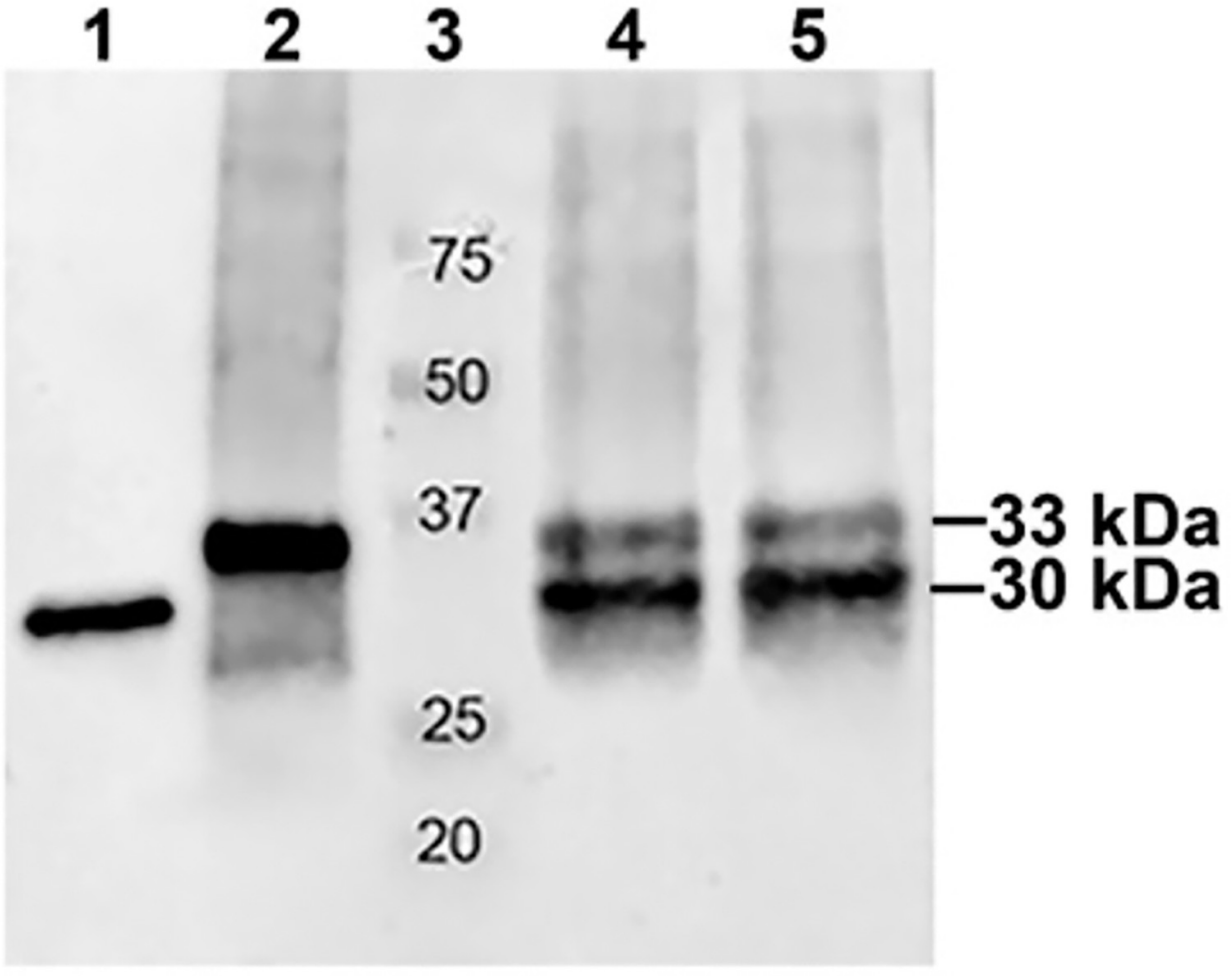
Western blot hybridized with affinity purified polyclonal antibodies to denatured *F*. *tularensis* rUsp/His_6_. Lane 1, 0.5 μg *F*. *tularensis* lysate. Lane 2, 0.01 μg purified *F*. *tularensis* rUsp/His_6_. Lane 3, protein molecular weight markers with sizes denoted in kilodaltons (kDa). Lanes 4 and 5, combined 0.5 μg *F*. *tularensis* lysate plus 0.01 μg *F*. *tularensis* rUsp/His_6_.

Western blot analyses showed that the endogenous *F*. *tularensis* Usp has an apparent molecular weight of ~30 kDa (Fig 1, lane 1), consistent with its theoretical molecular weight of 30188 Da. However, *F*. *tularensis* rUsp/His_6_ is larger at ~33 kDa (Fig 1, lane 2). The histidine tag accounts for 1 kDa, leaving unexplained an excess mass of 2 kDa. The salt in a protein sample can affect migration in an SDS gel. To be sure the difference in molecular weight is not an artifact of salt, the protein samples were mixed to produce a uniform salt concentration. Fig 1 lanes 4 and 5 show 2 bands for the mixed sample, thus ruling out the possibility that salt contributes to the higher molecular weight observed for rUsp/His_6_. It was concluded that the observed molecular weight difference is real and that *F*. *tularensis* rUsp/His_6_ expressed in *E*. *coli* is at least 3 kDa heavier than endogenous *F*. *tularensis* Usp.

These results demonstrated that the affinity purified rabbit polyclonal antibodies for denatured *F*. *tularensis* rUsp/His_6_ is specific for Usp, as indicated by the single band obtained for endogenous *F*. *tularensis* Usp in the lysate (Fig 1 lane 1). In addition, the ability to detect endogenous Usp in 0.5 μg of *F*. *tularensis* lysate suggests the Usp protein is abundant.

### Post-translational modifications on *F*. *tularensis* rUsp/His_6_

**Fig 2:**
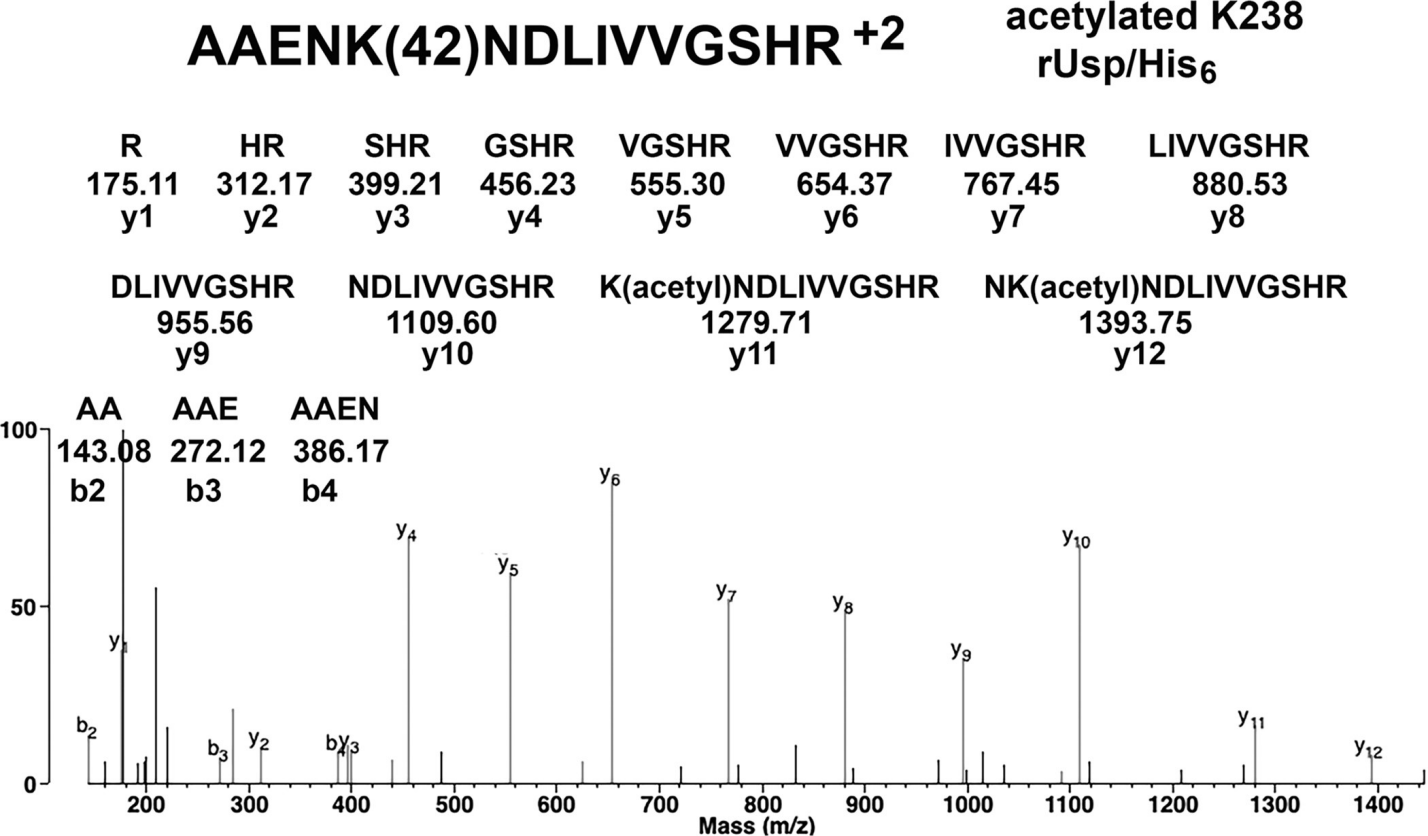
MS/MS spectrum for acetylated lysine 238 in *F*. *tularensis* rUsp/His_6_. The exact location of the 42.01 Da acetyl adduct is defined by the y1 to y10 ion series and the y11 ion whose structures are shown. The mass of the y11 ion at 1279.71 Da is 42.01 Da higher than the mass of the unmodified y11 ion. Masses of the y1 to y10 ions are consistent with the absence of an adduct on residues NDLIVVGSHR. The MH^+2^ parent ion has a mass of *m/z* 832.9394.

A possible explanation for the higher than expected molecular weight of the *F*. *tularensis* rUsp/His_6_ was initially postulated to be due to post-translational modifications. We used LC-MS/MS to generate fragmentation spectra of isolated rUsp/His_6_ peptides. The spectra were searched for acetylated and polyaminated adducts. Fig 2 presents convincing evidence for acetylated K238 in peptide AAENK(42)NDLIVVGSHR of rUsp/His_6_. Masses for the y11 and y12 ions are heavier by the 42 Da of the acetyl group, thus supporting acetylation of K238. The location of the 42 Da adduct is further defined by the y1 to y10 ions whose masses are free of 42 Da. Table 1 lists the peptides containing acetylated lysines in *F*. *tularensis* rUsp/His_6_. Of the 24 lysines in Usp, 10 are acetylated in rUsp/His_6_. It was estimated that less than 1% of any specific peptide was acetylated.

**Table 1:** Acetylated lysines in *E*. *coli* expressed *F*. *tularensis* rUsp/His_6_ (Accession # WP_003021757).

Peptide	Acetylated K	Score	% matched intensity
SIEQEAK_63_EALDK	K63+42.01	37.2	76.9
EALDK_68_LVTK	K68+42.01	36.0	96.0
LVTK_72_ISGIK	K72+42.01	36.2	92.5
AK_129_CDVLTVR	K129+42.01	31.0	90.6
DIAK_175_LYSAK	K175+42.01	24.9	71.9
TYETDK_201_VETTLDK	K201+42.01	42.0	95.1
VETTLDK_208_FAEK	K208+42.01	43.0	98.8
FAEK_212_NGITGEK	K212+42.01	44.4	97.5
SVMIGGISNSLLEK_233_A	K233+42.01	38.4	80.3
AAENK_238_NDLIVVGSHR	K238+42.01	55.5	96.2

Score is a statistic calculated by Protein Prospector. The higher the score, the more confidence in the assignment. A score of 20 or less is a poor score with little confidence in the assignment. A score of 30 or higher reflects high confidence in the assigned adduct. % matched intensity reflects the number and intensity of peaks that fit the assigned peptide. A 100% matched intensity means every peak in the spectrum is assigned to the peptide.

Marakasova [21] identified 280 acetylated proteins in *F*. *novicida* strain U112 by proteome analysis of proteins that had been enriched for acetylation by addition of glucose and acetate into the culture medium or by direct chemical acetylation of *N*^ε^-lysines in the cell lysate with acetyl phosphate. One of the acetylated proteins in this set was Usp. The Usp in *F*. *novicida* was acetylated on K180, K146, K219, and K212 [21]. Table 1 shows only acetylated K212 is common to both *F*. *novicida* U112 and *F*. *tularensis* Usp. Additionally, both SWISS-MODEL [22, 23] and AlphaFold [24, 25] 3D modeling of *F*. *tularensis* Usp (Q5NI44) indicated that K212 and the other nine acetylated lysines in Table 1 are exposed on the surface of the Usp protein.

**Fig 3:**
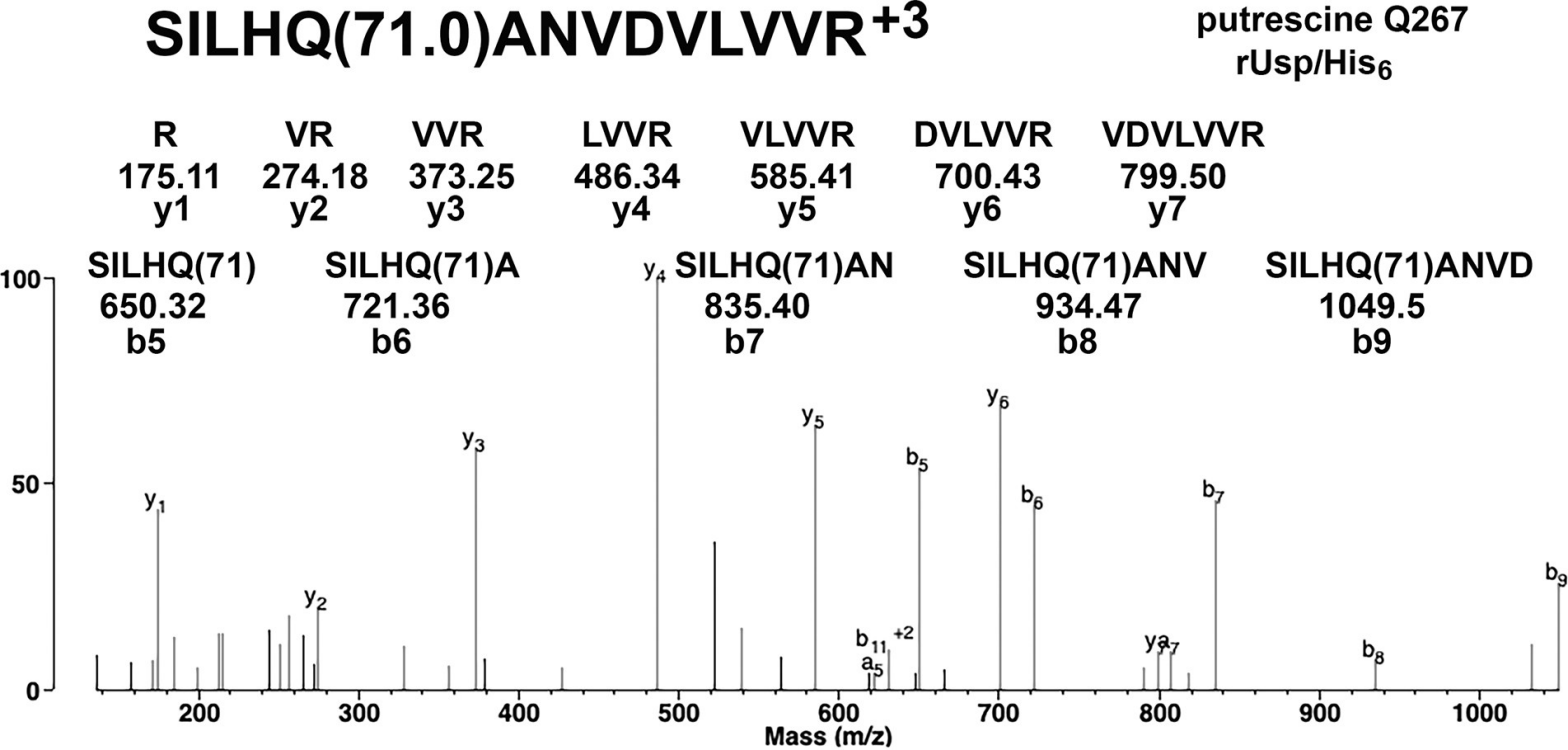
MS/MS spectrum for putrescine adduct on glutamine 267 of *F*. *tularensis* rUsp/His6. The b5 to b9 ions support putrescine on Q267 because they carry an added mass of 71 Da. The y-ions do not have an added mass of 71, which means putrescine is not on residues VDVLVVR. The MH^+3^ parent ion mass is m/z 545.3059. The parent ion minus a water molecule, MH-H_2_O^+3^, has a peak at m/z 539.2938.

The MS/MS spectrum in Fig 3 provides evidence for the presence of putrescine-modified glutamine 267 in *E*. *coli* expressed *F*. *tularensis* rUsp/His_6_. The putrescine adduct adds 71 Da to the masses of the b5 to b9 ions, whose structures are shown. Masses of the y1 to y7 ions define the amino acid sequence for an unmodified portion of the peptide.

**Fig 4:**
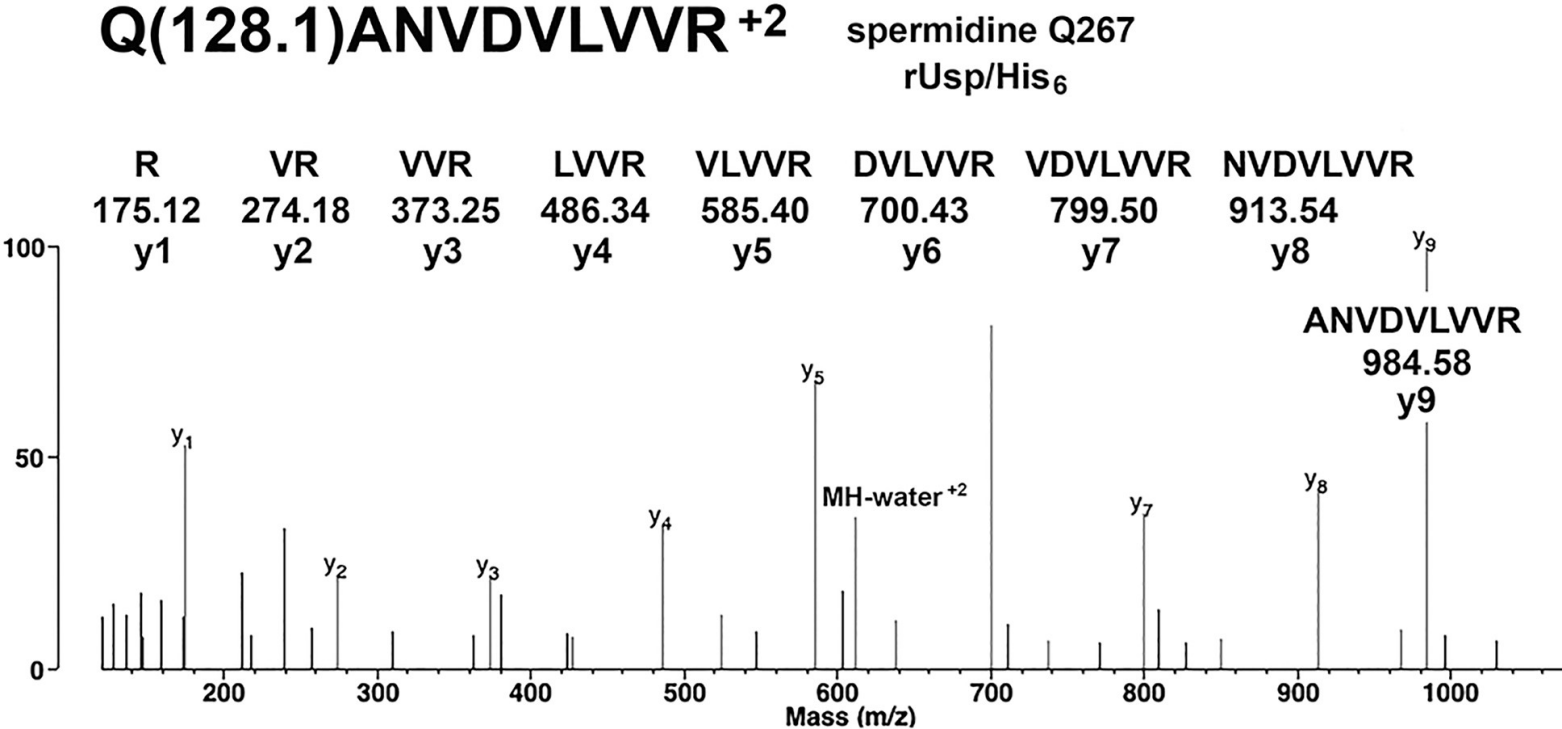
MS/MS spectrum for the spermidine adduct on Q267 of *F*. *tularensis* rUsp/His_6_. The structures of y1 to y9 ions are shown; none of the y-ions include the mass for spermidine. The added mass of 128.1 Da from spermidine is on glutamine. The MH^+2^ parent ion at m/z 620.8537 supports the presence of spermidine in this peptide. A peak for the parent ion minus water is at 611.8492 in charge state +2.

Evidence for a spermidine adduct on glutamine 267 in peptide QANVDVLVVR of *F*. *tularensis* rUsp/His_6_ is provided in Fig 4. The y-ions support the peptide sequence. None of the y-ions include the mass of spermidine. This leaves glutamine as the spermidine-modified residue. The mass of the parent ion supports the presence of the 128.1 Da spermidine in this peptide.

**Fig 5:**
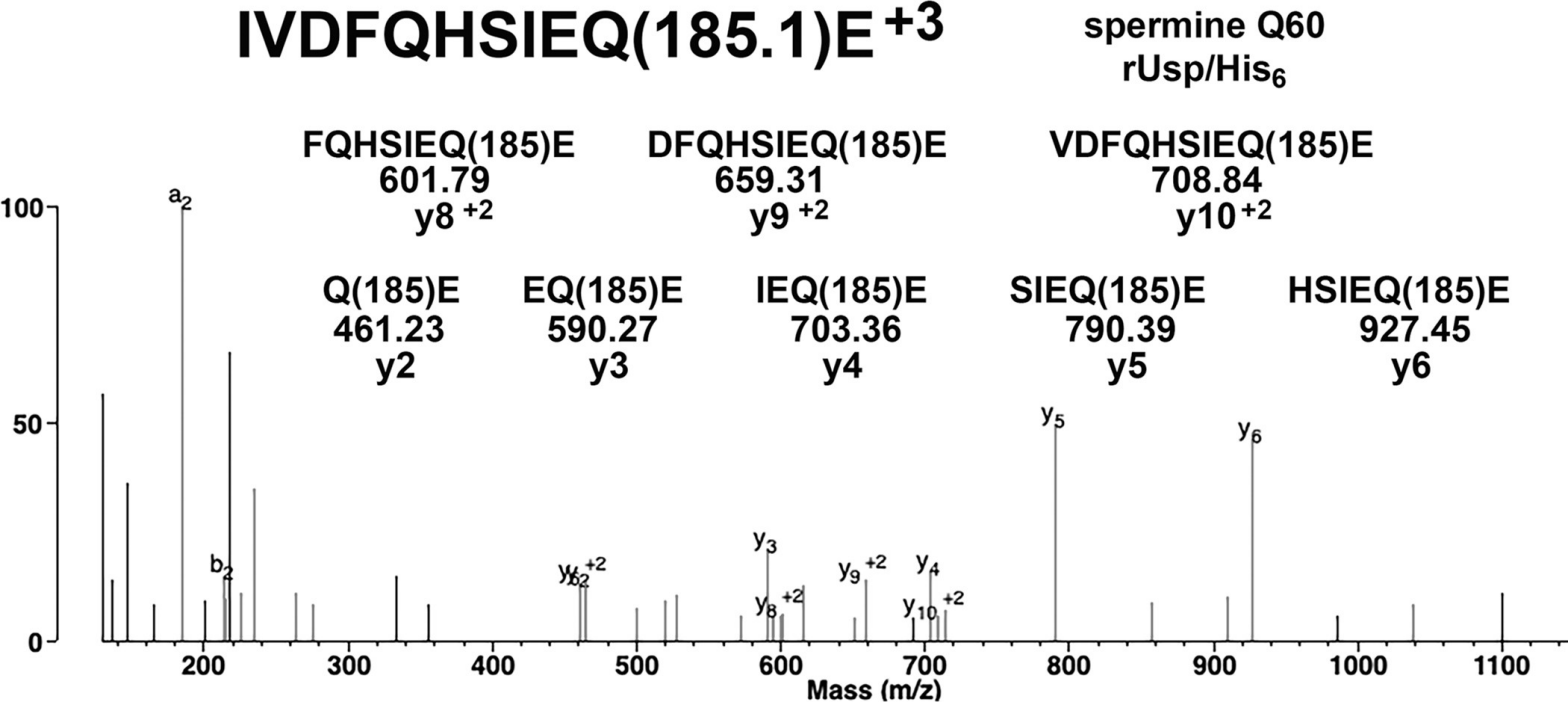
MS/MS spectrum for the spermine adduct on glutamine 60 of *F*. *tularensis* rUsp/His_6_. The structures of the y-ions show that all carry an added mass of 185 Da from spermine. The MH^+3^ parent ion is m/z 510.5917.

The MS/MS spectrum in Fig 5 supports the presence of a spermine adduct on glutamine 60 in peptide IVDFQHSIEQ(185.1)E of *F*. *tularensis* rUsp/His_6_. The masses of the y2 to y10 ions are consistent with an added mass of 185.1 Da on each of the structures shown. Table 2 summarizes the polyamine adducts identified by mass spectrometry in purified *F*. *tularensis* rUsp/His_6_. These findings provide evidence that a given glutamine such as Q267 can be modified by putrescine, spermidine, or spermine, suggesting that modifications fluctuate and are influenced by factors such as growth conditions.

**Table 2:** Polyamine adducts in *F*. *tularensis* rUsp/His_6_.

Peptide	Polyamine adduct	Score	% matched intensity
SILHQ_267_ANVDVLVVR	Q267 putrescine +71	35.6	86.1
VVTVIDCVAPFAPSIVDFQ_55_H	Q55 spermidine +128.1	47.6	92.0
ILHQ_267_ANVDVLVVR	Q267 spermidine +128.1	33.5	44.9
FFLGSTANSILHQ_267_AN	Q267 spermidine +128.1	45.4	88.2
FFLGSTANSILHQ_267_A	Q267 spermidine +128.1	42.4	85.2
LGSTANSILHQ_267_A	Q267 spermidine +128.1	31.8	62.1
Q_267_ANVDVLVVR	Q267 spermidine +128.1	28.5	64.9
APSIVDFQHSIEQ_60_EA	Q60 spermine +185.1	46.1	84.1
IVDFQHSIEQ_60_EA	Q60 spermine +185.1	25	72.6
FFLGSTANSILHQ_267_A	Q267 spermine +185.1	45.4	88.2

### Stop codon readthrough in *E*. *coli* expressed *F*. *tularensis* rUsp/His_6_

Although we found acetylated lysines and polyaminated glutamines, the greater than expected size of *F*. *tularensis* rUsp/His_6_ cannot be explained by post-translational modifications in *E*. *coli* since only a small percent of a given residue was modified. Even if each residue were 100% modified, the mass from the adducts would not add up to 3 kDa. Further, a ladder of bands would be expected if *F*. *tularensis* rUsp/His_6_ were a mixture of adducts with various masses, a result that was not observed. As depicted in Fig 1, immunoblot analysis consistently showed that *F*. *tularensis* rUsp/His_6_ migrated as a single band at 33 kDa, while endogenous *F*. *tularensis* Usp migrated as a single band at 30 kDa.

**Fig 6:**
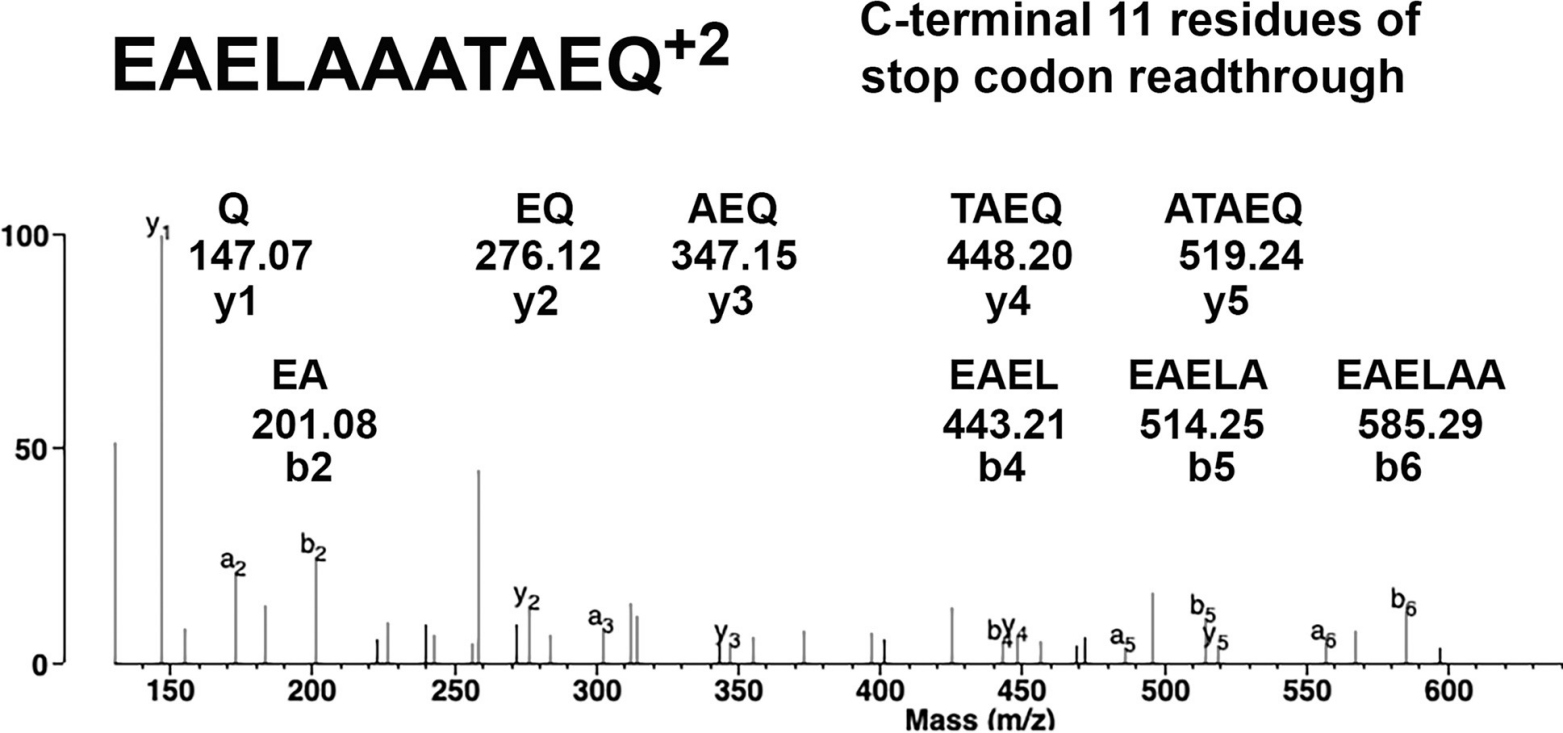
The 20 amino acid stop codon readthrough peptide, DPAANKARKEAELAAATAEQ, was contributed by the pET28a expression vector. Trypsin digestion of rUsp/His_6_ yielded the EAELAAATAEQ peptide. The b2-b6 ion series and the y1-y5 ion series in the MS/MS spectrum support the peptide sequence. The MH^+2^ parent ion is at m/z 552.2644. The stop codon readthrough is in frame with the histidine tag, adding a total of 28 amino acids to the C-terminus of rUsp/His_6_ via peptide LEHHHHHHDPAANKARKEAELAAATAEQ.

Our search for an alternative explanation led us to examine the possibility that *F*. *tularensis* rUsp/His_6_ comprised extra amino acids originating from the pET28 expression vector. When we updated the *F*. *tularensis* rUsp/His_6_ amino acid sequence to include residues from pET28, the mass spectrometry data confirmed that extra pET28 residues are present at the C-terminus. The C-terminus residues in *F*. *tularensis* rUsp/His_6_ included DPAANKARKEAELAAATAEQ, after the expected histidine tag (LEHHHHHH). Trypsin digestion of rUsp/His6 yielded peptide EAELAAATAEQ as shown in Fig 6. These findings demonstrated that a single TGA stop codon in the pET28 expression vector is not sufficient to terminate translation. The predicted molecular weight of *F*. *tularensis* rUsp/His_6_ containing the stop codon readthrough residues was 33.29 kDa, a mass consistent with the Western blot results (Fig 1).

## Discussion

### Mechanisms of post-translation modifications in bacteria

In bacteria, lysine acetylation occurs by two mechanisms: enzymatically with acetyltransferase and nonenzymatically with acetyl phosphate [26]. *E*. *coli* encode 26 acetyltransferases of which half have been experimentally validated to have enzymatic activity and are predicted to have different targets [27]. Acetylation is one of the mechanisms by which bacteria respond to stress, nutritional status, and inflammation [28], and can influence virulence and host-pathogen interactions [26, 29, 30]. Protein acetylation can occur on *N*^α^-amino groups such as the N-termini of proteins or on *N*^ε^-amino groups of lysine residues. *N*^α^-acetylation can tag the protein for degradation, which is often irreversible, whereas *N*^ε^-acetylation of lysine residues is reversible and dynamic, depending on the environment [27, 31]. *N*^ε^-acetylation of lysine residues can affect many protein functions, including enzymatic activity, stability, and protein- or DNA-protein interactions [32]. Nevertheless, most post-translational modifications are substoichiometric, which means that modifications are not present on all molecules of a given protein [30].

Polyaminated proteins are the product of the enzymatic activity of transglutaminase. Certain bacteria such as *Streptomyces mobaraensis* (formerly classified as *Streptoverticillium mobaraense*) secrete huge amounts of inactive pro-transglutaminase. Protease cleavage of an N-terminal peptide activates microbial transglutaminase [33]. Active transglutaminase can polyaminate and crosslink proteins. The crosslinking activity of microbial transglutaminase is used in the production of sausages, ham, yogurt, cheese, imitation crab and other foods [34]. *E*. *coli* strain K-12 is not known to have transglutaminase activity, although the UniProt database lists four entries for transglutaminase-like domain-containing proteins. Hemolytic strains of *E*. *coli* do have transglutaminase activity in a cytotoxic necrotizing factor that activates small Rho GTPases by deamidating Gln 63 [35]. Our report of polyaminated recombinant rUsp/His_6_ protein provides evidence for low levels of transglutaminase activity in *E*. *coli* strain BL21(DE3).

Active transglutaminase in bacteria generally is associated with the bacterial cell wall, where its crosslinking activity strengthens the cell wall. However, transglutaminase activity is tightly controlled. Uncontrolled transglutaminase activity in an *E*. *coli* expression system was lethal to the bacteria due to excessive transglutaminase catalyzed protein crosslinking [33, 36].

### Mechanisms of stop codon readthrough in bacteria

Proper termination of protein synthesis occurs at UAA, UAG, and UGA stop codons, and is catalyzed by release factors [37]. Stop codon readthrough has been described for proteins expressed in both bacteria and eukaryotes [11]. One mechanism that contributes to stop codon readthrough is due to suppressor tRNAs that carry mutations in the anticodon for the relevant stop codon [38]. Environmental and metabolic stress substantially increase the level of stop codon readthrough during protein synthesis in bacteria, promoting translational heterogeneity and phenotypic diversity [14]. However, since the rUsp/His_6_ protein has a single, defined mass of 33 kDa, the cause of stop codon readthrough is not likely explained by stress.

The pET28 plasmid has TGA in the position of the readthrough stop codon, which follows the histidine tag (see Addgene web page https://www.addgene.org/browse/sequence_vdb/2565/). The TGA stop codon is susceptible to readthrough [11, 12]. Therefore, the use of TGA and other factors (i.e., mRNA structural features) during translation apparently contributed to stop codon readthrough in *E*. *coli*.

### Evaluation of protein prospector search results

We rejected most of the computer-generated candidates for adducts because they did not provide convincing evidence for the adduct. Acceptable spectra have a Matched Intensity in the 60 to 100% range. We rejected a candidate when we could fit unassigned peaks in the MS/MS spectrum to an alternative amino acid sequence. The most convincing MS/MS spectra have ions on both sides of the modified residue as in Fig 2 for acetylated K238 in *F*. *tularensis* rUsp/His_6_, and no peaks that can be assigned to an alternative amino acid sequence.

Even though proteins were digested with a high-quality trypsin, nonspecific cleavage occurs. We find more peptides with a NO ENZYME search than with a TRYPSIN search. Peptides whose C-terminal is not positively charged, such as Glu (E) in Fig 5 do not fragment to yield y1 ions, in all likelihood because the amino acid at this position does not support a positive charge. Nevertheless, we accept the spermine (+185 Da) adduct on Q60 of *F*. *tularensis* rUsp/His_6_ in Fig 5 because the added mass of +185 Da on y2 to y10 ions strongly supports the spermine (+185 Da) adduct. In addition, there are no unassigned peaks in this spectrum to offer an alternative interpretation.

### Examples of rejected hits

Protein Prospector suggested Q33 of *F*. *tularensis* rUsp/His_6_ was modified by spermidine in peptide NNTQ(128.1)VLKVVTVIDCVAPFAPSIVDFQHSIEQEAK. The matched series intensity was 100%, which means all peaks in the MS/MS spectrum were assigned to the modified peptide. Supporting b- ions ranged from b5 to b13, but there were no b1, b2, b3, or b4(128) ions. As it turned out, the residue to the N-terminal side of the peptide is K. A missed cleavage would yield peptide KNNTQVLKVVTVIDCVAPFAPSIVDFQHSIEQEAK. The dehydro-mass of K is 128 Da. Therefore, the mass of KNNTQ is equal to the mass of NNTQ(128). Both the adduct and the missed cleavage peptide fit the MS/MS spectrum and have the same parent ion mass of m/z 3925.05. This ambiguity in interpretation led us to reject this candidate.

**Fig 7:**
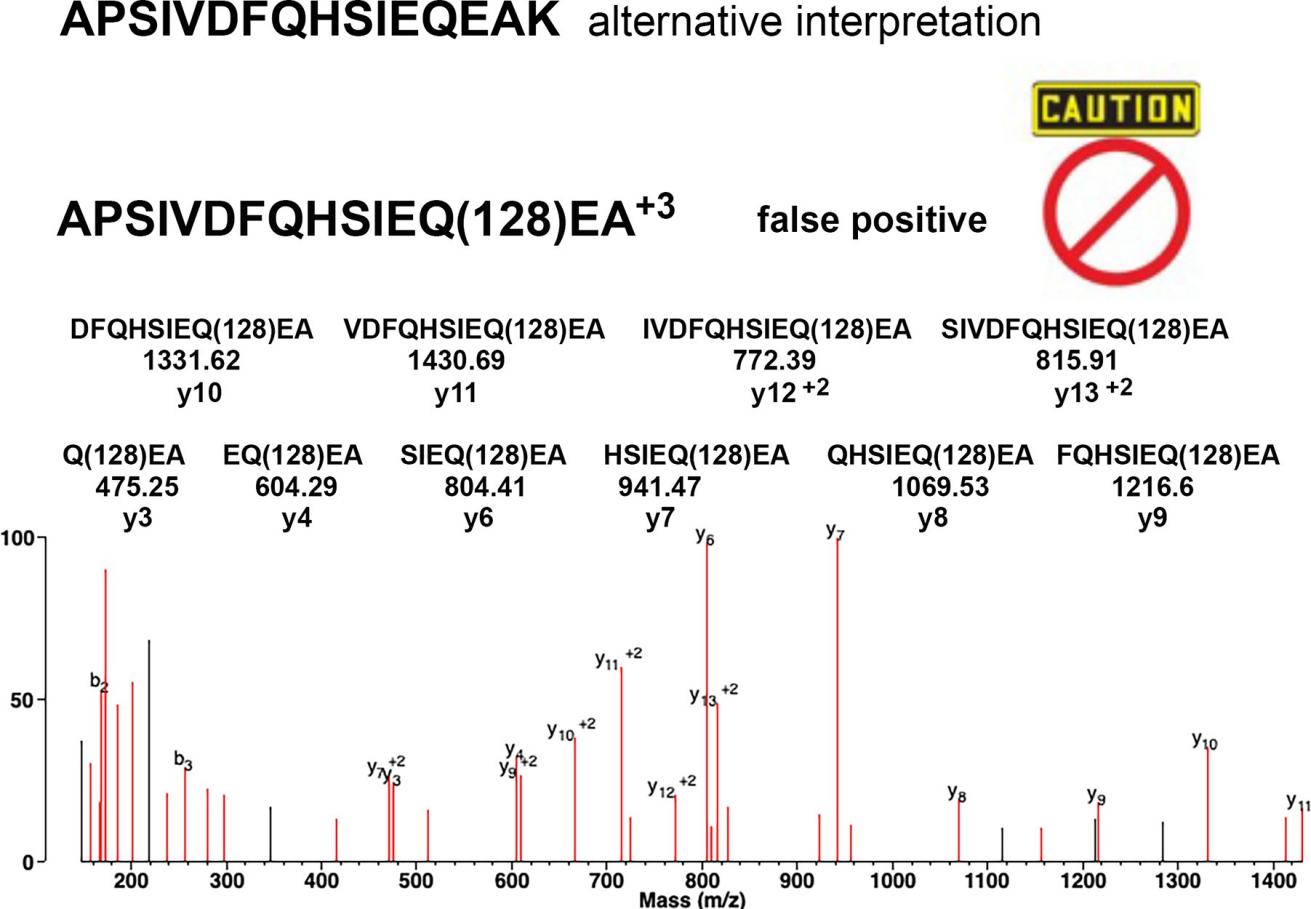
False positive spermidine adduct (+128) on Q60 of rUsp/His6. On first inspection the y3 to y13 ion series, the score of 50.2 and the matched intensity of 89% gave strong support for a spermidine adduct on Q60. However, the absence of y1 and y2 ions leaves open an alternative interpretation. Lysine and spermidine have the same 128 Da mass. The parent ion for the spermidine adduct above is m/z MH^+3^ 600.3044 and that of the Usp peptide APSEIVDFQHSIEQEAK, including K at the C-terminus, is almost identical at m/z 600.3039. It was concluded that the evidence for a spermidine adduct on Q60 is inadequate. The candidate adduct was therefore rejected.

Spermidine modified Q60 in peptide APSIVFQHSIEQ(128.1)EA of rUsp/His_6_ was rejected despite the presence of supporting y3 to y13 ions. The MS/MS spectrum in Fig 7 has no y1 or y2 ions. It was noticed that an added K at the C-terminus adds 128 Da. The MS/MS spectrum had actually identified APSIVFQHSIEQEAK. The 128 Da mass was from K and not from spermidine.

## Supporting information

S1 Raw images Uncropped, unadjusted Western blot in text as Fig 1. (DOCX)

S1 Graphical abstract (TIF)

## Abbreviations

Usp: universal stress protein
rUsp/His_6_: recombinant universal stress protein with a C-terminal histidine tag
LC-MS/MS: liquid chromatography tandem mass spectrometry
