# Supplementary material for: Polyaminated, acetylated and stop codon readthrough of recombinant Francisella tularensis universal stress protein in Escherichia coli
Source: PLoS One. 2024 Apr 29;19(4):e0299701. doi: 10.1371/journal.pone.0299701 (PMC11057771; doi:10.1371/journal.pone.0299701)
Supplement: S1 Raw images — (DOCX) [file pone.0299701.s001.docx]

Supporting Information

Polyaminated, acetylated and stop codon readthrough of recombinant *Francisella tularensis* Universal stress protein in *Escherichia coli*

Benjamin Girardo^1^, Lawrence M. Schopfer^2^, Yinshi Yue^1^, Oksana Lockridge^2*^, Marilynn A. Larson^1^

^1^ Pathology and Microbiology Department, University of Nebraska Medical Center, Omaha, NE, USA

^2^ Eppley Institute, University of Nebraska Medical Center, Omaha, NE, USA

| 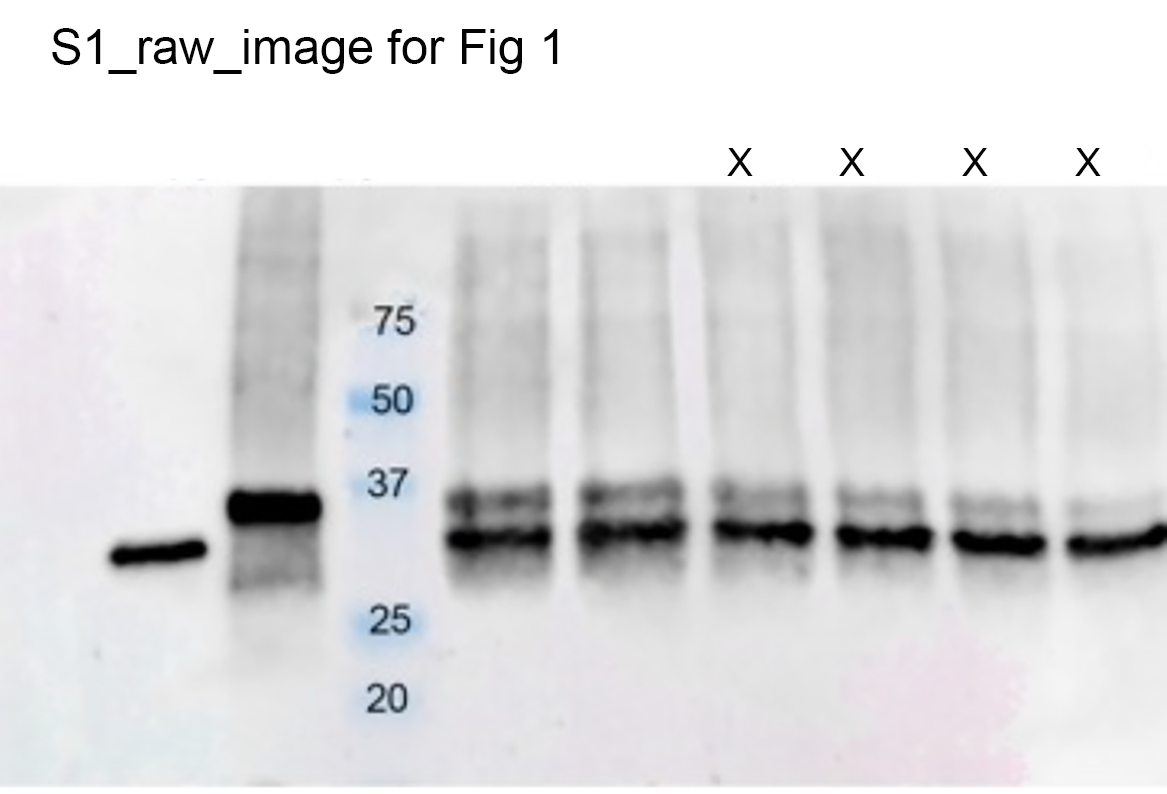 |
| --- |

S1_raw_images. Uncropped, unadjusted Western blot of figure 1 in the text. This blot complies with the requirement that authors provide the original uncropped and unadjusted images underlying all blot or gel results reported in a submission’s figures or Supporting Information files.

The image was acquired on an Azure 600 imaging device.
