# Supplementary figures and images for: Polyaminated, acetylated and stop codon readthrough of recombinant Francisella tularensis universal stress protein in Escherichia coli
Source: PLoS One. 2024 Apr 29;19(4):e0299701. doi: 10.1371/journal.pone.0299701 (PMC11057771; doi:10.1371/journal.pone.0299701)

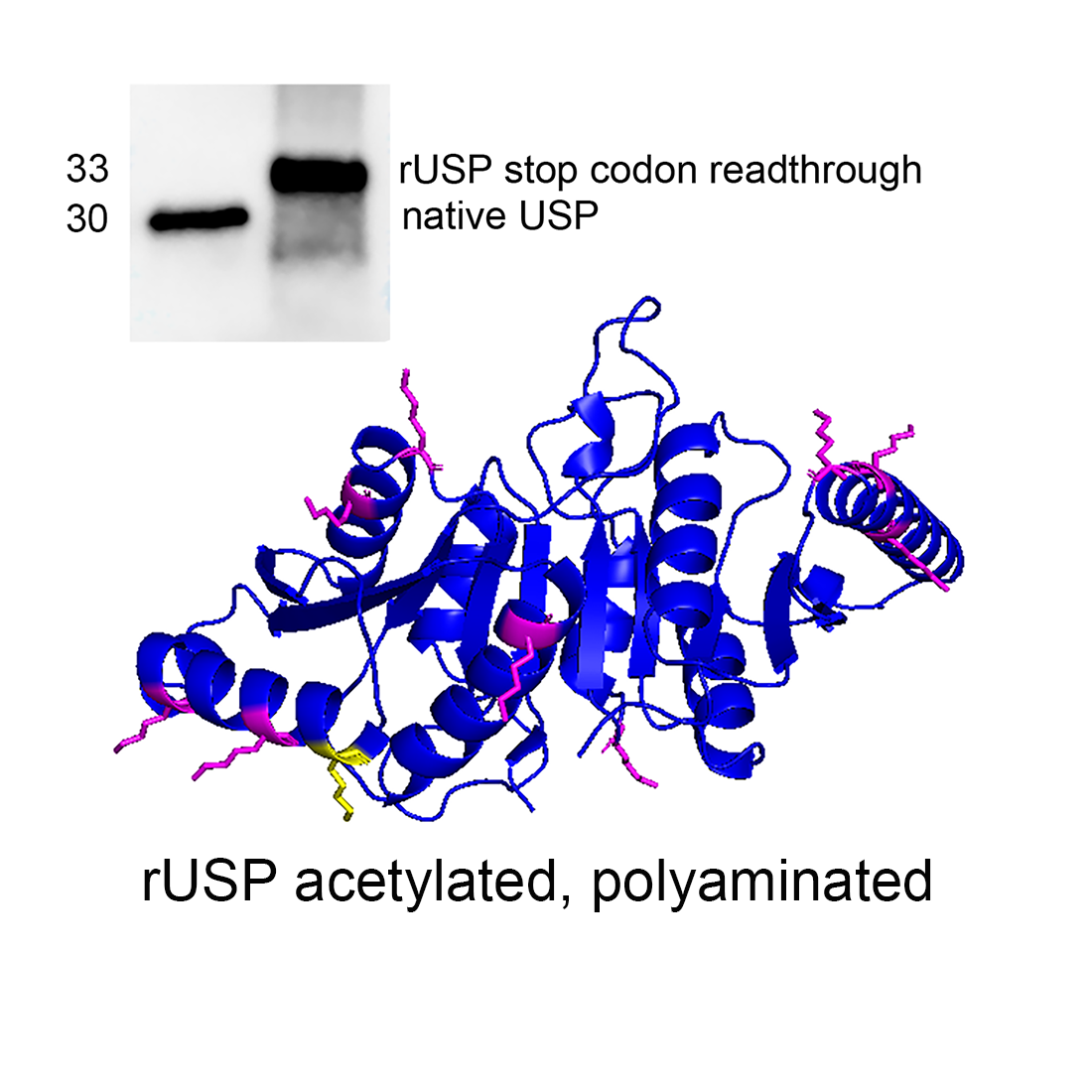

Supplement: S1 Graphical abstract — (TIF) [file pone.0299701.s002.tif]
